# Editorial: Quantifying and controlling the nano-architecture of neuronal synapses

**DOI:** 10.3389/fnsyn.2022.1024073

**Published:** 2022-09-07

**Authors:** Xiaobing Chen, Thomas Kuner, Thomas A. Blanpied

**Affiliations:** ^1^Laboratory of Neurobiology, National Institute of Neurological Diseases and Stroke, National Institutes of Health, Bethesda, MD, United States; ^2^Department of Functional Neuroanatomy, Institute for Anatomy and Cell Biology, Heidelberg University, Heidelberg, Germany; ^3^School of Medicine, University of Maryland, Baltimore, MD, United States

**Keywords:** synapse, nano-architecture, dynamics, super-resolution imaging, volume EM, EM tomography, correlated microscopy

About 2 years ago when the journal gave us the opportunity to launch this Research Topic, the three of us were excited by the rapid growth of cutting-edge imaging approaches now illuminating the architecture and molecular organization of synapses. Since its launch, we received enthusiastic responses from many authors and in the end, we published 15 articles in this volume.

These articles, including original research, reviews, and opinion, present a snapshot of several key active areas of ongoing work. They fall roughly into three areas: technical advances in single-molecule imaging and analysis, dynamics of molecular organization within the synapse, and advances in electron microscopy that are propelling new insights.

## Technical advances in single-molecule imaging and analysis

This group of papers illustrates well how light-based super-resolution imaging is providing important new routes to visualize and analyze the distribution of proteins in single synapses. Particularly including single-molecule, expansion microscopy, and SIM. New methods are achieving ever-better resolution while also making these technically challenging approaches easier to use by more laboratories. Here, Unterauer and Jungmann provided an updated review of DNA PAINT technology which provides single-nanometer resolution with straightforward routes to molecular counting along with highly multiplexed imaging. A great advantage of the approach is that with a single labeling step, antibodies conjugated with short DNA oligonucleotides can label multiple targets that are subsequently imaged by sequential exchange of fluorophore-labeled complementary oligonucleotides, avoiding chromatic aberration. Narayanasamy et al. then further expanded the use of DNA-PAINT by demonstrating multiplexed imaging of multiple pre- and post-synaptic proteins in brain slices, demonstrating the viability of this important approach for analyzing synaptic nanostructure *in vivo*. In a Perspective, Specht argued that single-molecule imaging provides a powerful approach to determining protein copy number, providing both high throughput and high subcellular spatial resolution to interpret such counts. Copy number of key postsynaptic density (PSD) proteins have been studied using calibrated fluorescent light microscopy (Sugiyama et al., 2005) or scanning transmission electron microscopy (STEM) (Chen et al., 2005), and these numbers have been used to estimate the copy number of many other PSD proteins using quantitative mass spectrometry (Cheng et al., 2006; Martinez-Sanchez et al., 2021) and a recent study demonstrated estimation of protein number copy in individual spines by coupling super-resolution imaging and quantitatively mass spectrometry (Helm et al., 2021). New single-molecule methods are likely to offer an opportunity to map changes of the copy number of key synaptic proteins during synaptic activity.

Several new advances within this arena were also introduced in this Topic. Kuhlemann et al. demonstrated site-specific labeling of the extracellular domain of γ-aminobutyric acid type A (GABA_A_) receptor subunits by genetic code expansion with unnatural amino acids combined with bio-orthogonal click-chemistry labeling with tetrazine dyes. Gagliano et al. provided a comprehensive review of single molecular tracking and super-resolution imaging of synaptic proteins in 3D enabled by new light sheet illumination approaches. Sneve and Piatkevich provided an overview of new insights that may arise from using expansion microscopy to map synapses in neuronal circuits. These contributions highlight the enormous potential of combining novel labeling approaches with novel microscopy techniques.

## Dynamics of molecular organization within the synapse

A second group of papers focused on new aspects of molecular dynamics that support synaptic functional diversity. Piao and Sigrist provided a detailed review of work from Drosophila which shows how the active zone protein (M)Unc13 defines the behavior of individual release sites of the active zone. More generally, the work illustrates that isoform-specific components of the vesicle release machinery may drive functional presynaptic heterogeneity, revealing principles likely at play in the mammalian brain as well. In new research, Maschi et al. imaged release of individual vesicles (Tang et al., 2016; Maschi and Klyachko, 2017) to determine that Myosin V regulates both temporal and spatial utilization of release sites during two main forms of synchronous release in the presynaptic active zone. Using a modeling approach, they describe Myosin V function as controlling a gradient of release site release probability across the active zone, thereby uniting spatial and temporal functions of Myosin V in uni-vesicular and multi-vesicular release. Westra et al. reviewed our current understanding of how several lipids specifically enriched the synapse are organized at the synaptic membrane. They bring together compelling arguments that organization of the membrane itself in fact could contribute to protein distribution at the synapse and to synaptic transmission. They conclude with a call for new technologies to define and test the influence of synaptic lipid nanoscale organization of synaptic proteins.

In recent years, many studies using super-resolution light microscopy techniques including PALM, dSTORM, single-molecule tracking, STED, SIM, and Expansion Microscopy have found that key scaffolding proteins in the excitatory PSD such as PSD-95, and receptors such as NMDARs and AMPARs, are located subsynaptically in areas of high density termed nanoclusters or nanodomains (Fukata et al., 2013; MacGillavry et al., 2013; Nair et al., 2013; Broadhead et al., 2016; Tang et al., 2016; Goncalves et al., 2020). This work has focused attention on the potential that discrete adhesion molecule systems may control transsynaptic alignment (Haas et al., 2018; Ramsey et al., 2021). Expanding this investigation in inhibitory synapses, Gookin et al. report synaptic nanoclusters of the adhesion protein neuroligin-2, which are arrayed similarly to nanoclusters of the GABA_A_R (Crosby et al., 2019), suggesting an important role in establishing synapse nanoarchitecture. Through direct quantitative comparison of super-resolution methods, they report further that dSTORM provided a more detailed view of the protein density landscape of neuroligin-2. This is an important observation in part because combining nanostructure with trafficking and diffusion dynamics of proteins is a rich area for incoming work. A powerful approach to this general problem applied specifically to the case of adhesion molecules like the neuroligins is provided by Lagardère et al. who demonstrated the use of FluoSIM, a new simulator of membrane protein dynamics for fluorescence microscopy. Correlating high-resolution imaging experiments with simulations of Neurolin1 at synaptic vs. extrasynaptic sites enabled biological and biophysical interpretation of the imaging data, and advanced new ideas about how Neuroligin-Neurexin interactions play essential roles in organizing the synaptic cleft.

Emphasized by all these findings, it is clear that further exploration is still needed of how subsynaptic protein nanoclusters/nanodomains govern synapse function. The importance of this undertaking is even further emphasized by work here from Sun et al., who used dSTORM to image the nanometer-scale trans-synaptic alignment of key proteins in the active zone and PSD during synaptic development and maturation. They report that despite profound changes in the abundance and nanoarchitecture of these proteins across early development in culture—or even following prolonged, total blockade of neuronal activity—transcellular protein nano-alignment remained robust.

## Progress in electron microscopy of synapses

The field of electron microscopy is teeming with technical advances in microscopy (e.g., significantly larger, faster, and more sensitive electron detection cameras, much better optics and much more stable stages and more efficient computer-driven automated image acquisition schemes), new and improved sample preparation techniques and advances in image processing software development are all powering new discoveries.

Here we collected three EM related papers as vignettes of this blooming field. To further dissect structural basis of synaptic transmission, information of subsynaptic structures/organelles now often need to be quantified in specifically designed experiments. Watanabe et al. reported their development of sets of image analysis programs for aiding quantitative morphometric analysis (e.g., location and number) of subcellular organelles such as vesicles, endosomes in synaptic terminals, this would be especially useful for large bulk of serial section EM images where manually analysis might be too daunting and unproductive; they also introduce a scheme to address sampling biases in image analysis, a much needed feature to have.

Petralia et al. provided a review of a class of underappreciated structures called invaginations, small outward projections from one cell membrane to another in various synaptic junctions, which is often too small to be seen by light microscopy and was often misidentified by traditional thin section EM. Invaginations appear to be important for further understanding processes in synaptic development, maintenance, and plasticity. Now, focused ion beam-scanning electron microscopy (FIB-SEM), one of the new 3D EM or volume EM methods, makes tracing and detailed analysis of these invaginating structures possible.

3D volume EM uses fixed, heavy metal-stained, and plastic-embedded brain tissues imaged by serial block-face scanning EM (SBEM), requiring either mechanical serial sectioning (Denk and Horstmann, 2004) or FIB milling (Knott et al., 2008; Wu et al., 2017; Xu et al., 2021) and imaging with back-scattered electrons. Larger sample volumes are imaged by the automated tape-collecting ultramicrotome (ATUM), where ~30 nm thick serial sections are imaged by SEM (Kasthuri et al., 2015) or by transmission EM (Yin et al., 2020) to allow reconstruction and mapping of thousands of synapses in the neuropil (Hua et al., 2015). The resulting huge 3D datasets requires machine learning based automated segmentation (Heinrich et al., 2021). For volumes < ~100 cubic micrometers, thick-section bright field scanning transmission EM (STEM) tomography of 1–2 μm thick plastic embedded sections provide 3–4 nm isotropic resolution without sectioning resulted in reconstructions of entire ribbons in rat rod bipolar cell ribbon synapses (Graydon et al., 2014) and smaller spines or entire PSDs (Chen et al., 2014, 2015) in rat hippocampal cultures. Another useful technique is the development of the conjugate light and EM array tomography (Collman et al., 2015), which enables mapping the transmitter types in large numbers of synapses in brain tissue in great detail.

Finally, Szule provided detailed hypotheses of molecular identities of various structures in the regular array of a large macromolecular assembly, the active zone material, a dedicated molecular machinery responsible for vesicle retention, delivery and fusion at the presynaptic terminal of the frog neuromuscular junction (NMJ). The organization of these active zone materials and its structural model was based on 3D reconstructions from EM tomography of fixed or freeze-substituted frog NMJ. A molecular level structural model detailing vesicle fusion processes at the presynaptic terminal of the NMJ active zone might have broader implications for understanding the molecular basis of synaptic transmission in other types of synapses in general. Super-resolution light microscopy is now revealing the molecular organization of active zone proteins at NMJ (Badawi and Nishimune, 2020). It would be of great interest to see a unified molecular structural model based on EM and LM in the near future.

Transmission EM tomography of synapses has allowed the creation of 3D reconstructions to delineate organization of subsynaptic organelles, key synaptic proteins, and macromolecular complexes at pre and postsynaptic terminals in fixed or high pressure frozen and freeze-substituted neuronal culture, brain slice culture or nerve tissue (Harlow et al., 2001; Chen et al., 2008; Burette et al., 2012; Watanabe et al., 2013; Imig et al., 2014) and in vitrified hydrated synapses in neuronal cultures or isolated synaptosomes using cryo-EM tomography (Zuber et al., 2005; Fernandez-Busnadiego et al., 2013; Tao et al., 2018; Martinez-Sanchez et al., 2021). Structures reconstructed by EM tomography provide size, shape, and location of protein complexes, subcellular organelles in synapses, but at current 2–4 nm resolution, cannot guarantee unambiguous molecular identification of individual structures at synapses. Matching the size and shape of structures in tomograms to high resolution structures of individual proteins such as PSD-95 MAGUKs (Nakagawa et al., 2004) or extracellular domains of glutamate receptors (Nakagawa et al., 2005; Sobolevsky et al., 2009) may lead to molecular identity. Efforts were made to combine immunogold labeling (Chen et al., 2008, 2011) or miniSOG (Chen et al., 2018) with EM tomography to identify a certain class of structures at synapses such as PSD-95 as vertical filaments at the PSD. In other cases, KO animals (Fernandez-Busnadiego et al., 2013; Imig et al., 2014; Schrod et al., 2018) or protein knockdown (Chen et al., 2011, 2015) were combined with EM tomography to test hypotheses regarding molecular identity of classified structures. Recent work using averaging techniques in cryo EM tomograms (Liu et al., 2020; Martinez-Sanchez et al., 2021) showed that individual receptors at synapses might be identifiable by their extracellular domain morphology. Finally, it will be exciting to see how two major approaches to molecular nanostructure, direct imaging of proteins in cryo-fixed samples using cryo-ET combined with subsequent identification of protein identity vs. imaging of fluorescently labeled proteins using super-resolution microscopies, with MINFLUX (Balzarotti et al., 2017) reaching nanometer resolution, will converge on synaptic nanoarchitecture.

Together, the articles of this Research Topic highlight that synaptic nanoarchitecture is a blossoming field helping to grow and propagate important technical advances and unearthing new insight to synaptic function. We close this volume of the topic with hope that we will come back to this lively garden of ours in due time to welcome more papers that highlight exciting new progress and development in this vibrant field. We look forward to seeing you then!

## Author contributions

XC, TK, and TB conceived the main ideas and wrote the manuscript. All authors contributed to the article and approved the submitted version.

## Funding

XC is supported by the NINDS intramural program. TB is supported by R37MH080046. TK is supported by grants of the Baden-Württemberg Stiftung.

## Conflict of interest

The authors declare that the research was conducted in the absence of any commercial or financial relationships that could be construed as a potential conflict of interest.

## Publisher's note

All claims expressed in this article are solely those of the authors and do not necessarily represent those of their affiliated organizations, or those of the publisher, the editors and the reviewers. Any product that may be evaluated in this article, or claim that may be made by its manufacturer, is not guaranteed or endorsed by the publisher.
